# N_**2**_ Gas Plasma Inactivates Influenza Virus by Inducing Changes in Viral Surface Morphology, Protein, and Genomic RNA

**DOI:** 10.1155/2013/694269

**Published:** 2013-09-30

**Authors:** Akikazu Sakudo, Naohiro Shimizu, Yuichiro Imanishi, Kazuyoshi Ikuta

**Affiliations:** ^1^Department of Virology, Research Institute for Microbial Diseases, Osaka University, Yamadaoka, Suita, Osaka 565-0871, Japan; ^2^Laboratory of Biometabolic Chemistry, School of Health Sciences, University of the Ryukyus, Nishihara, Okinawa 903-0215, Japan; ^3^NGK Insulators, Mizuho-ku, Nagoya 467-8530, Japan

## Abstract

We have recently treated with N_2_ gas plasma and achieved inactivation of bacteria. However, the effect of N_2_ gas plasma on viruses remains unclear. With the aim of developing this technique, we analyzed the virucidal effect of N_2_ gas plasma on influenza virus and its influence on the viral components. We treated influenza virus particles with inert N_2_ gas plasma (1.5 kpps; kilo pulses per second) produced by a short high-voltage pulse generated from a static induction thyristor power supply. A bioassay using chicken embryonated eggs demonstrated that N_2_ gas plasma inactivated influenza virus in allantoic fluid within 5 min. Immunochromatography, enzyme-linked immunosorbent assay, and Coomassie brilliant blue staining showed that N_2_ gas plasma treatment of influenza A and B viruses in nasal aspirates and allantoic fluids as well as purified influenza A and B viruses induced degradation of viral proteins including nucleoprotein. Analysis using the polymerase chain reaction suggested that N_2_ gas plasma treatment induced changes in the viral RNA genome. Scanning electron microscopy analysis showed that aggregation and fusion of influenza viruses were induced by N_2_ gas plasma treatment. We believe these biochemical changes may contribute to the inactivation of influenza viruses by N_2_ gas plasma.

## 1. Introduction

Infection mediated by medical devices is thought to be a major contributor to hospital-acquired infections [1]. However, medical devices and instruments are often not sufficiently robust to withstand repeated rounds of sterilization by autoclaving or dry-heat treatment [2]. Alternative sterilization techniques involve the generation of *γ*-rays or electron beams, which require expensive facilities and are not appropriate for routine daily use [3]. Although ethylene oxide gas (EOG) can be used to sterilize heat-sensitive medical instruments, the gas is both toxic and carcinogenic, which limits its usage [4]. Recently, sterilization using hydrogen peroxide gas plasma was proposed, although it is ineffective against endotoxins and lipopolysaccharides (LPSs) [5, 6]. Residual amounts of endotoxin derived from bacteria may cause symptoms including fever [7].

A gas plasma is generated by removing electrons from a gas to produce a highly excited mixture of charged nuclei and free electrons [8, 9]. Recently, we succeeded in generating N_2_ gas plasma using a fast high-voltage pulse from a static induction (SI) thyristor power supply [6, 8–10]. N_2_ gas plasma treatment efficiently inactivates bacteria and bacterial spores, as well as degrading LPS, which showed a more than 5 log reduction in 30 min [6]. The *D* value (the decimal reduction time) of *Geobacillus stearothermophilus* was less than 1.3 minutes, whereas that of *Aspergillus niger* was even smaller [6]. However, the effect of N_2_ gas plasma on viruses remains unclear. Therefore, we treated influenza virus, as a representative enveloped virus, with N_2_ gas plasma and analyzed the sterilizing efficiency. We also analyzed the effect of this treatment on viral components such as proteins and RNAs.

## 2. Materials and Methods

### 2.1. Viruses Nasal Aspirates

Nasal aspirates were collected from children at the Baba pediatric clinic (Kadoma, Osaka, Japan) as described previously [11]. Briefly, saline was introduced into the nasal cavity, and then the wash solution was aspirated using Belvital (Melisana, Nogent-sur-Marne, France). In order to remove cell debris, the nasal fluid was filtered using a stainless steel mesh [200 grids per inch (25.4 mm)]. The obtained nasal aspirates were checked by immunochromatography for influenza viruses A and B as described below. Each aspirate sample (3 influenza A and 3 influenza B) was subjected to analysis. The research project for respiratory infectious diseases was approved by the Ethics Committees of the Research Institute for Microbial Diseases in Osaka University, and written informed consent was obtained from the patients.

### 2.2. N_2_ Gas Plasma Instrument and Treatment

**Figure 1:**
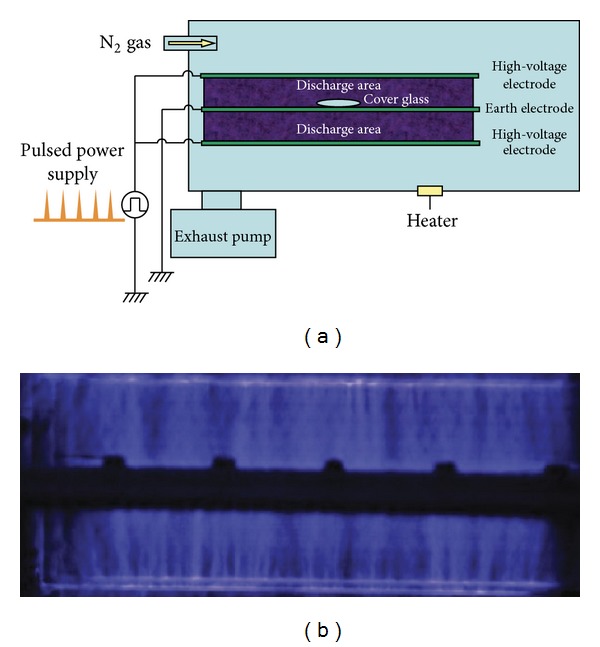
Experimental setup for N_2_ gas plasma production. (a) Schematic of N_2_ gas plasma instrument (BLT-TES No.1) used in this study. The distance between the high-voltage electrode and the earth electrode was 50 mm. N_2_ gas flow rate was 10 L/min. During N_2_ gas plasma generation, the sample box was kept at 0.5 atmospheric pressure. (b) Photograph of discharge region during generation of N_2_ gas plasma. A static induction (SI) thyristor was used as a pulsed power supply.

BLP-TES No.1 (NGK Insulators, Ltd) was used as a device to produce N_2_ gas plasma by generating a fast high-voltage pulse utilizing a SI thyristor power supply (Figure 1). The sample was exposed to N_2_ gas (99.9995%, Okano, Okinawa, Japan) at about 0.5 atmospheres prior to applying the high-voltage pulse. A 20 *μ*L aliquot of each sample solution was dropped onto a cover glass, air dried, and then treated with N_2_ gas plasma at 1.5 kpps (kilo pulses per second). Each sample on the cover glass was subsequently recovered by dissolving in 20 *μ*L of distilled water (Otsuka Pharmaceuticals Co., Tokyo, Japan).

### 2.3. Allantoic Fluid

Influenza virus-infected allantoic fluid was prepared from 11-day-old chicken embryonated eggs injected with influenza A virus H1N1 (A/PR/8/34) or influenza B virus (B/Gifu/2/73).

### 2.4. Purified Influenza A and B Viruses

Allantoic fluids of 11-day-old embryonated eggs infected with either influenza A virus H1N1 (A/Taiwan/1/86), H3N2 (A/Panama/2007/99), or influenza B virus (B/Tokio/53/99) were collected after 48 h incubation at 37°C and subjected to fractionation using a sucrose density gradient generated by ultracentrifugation. The obtained fraction was checked by sodium dodecyl sulfate (SDS) polyacrylamide gel electrophoresis (PAGE) followed by Coomassie Brilliant Blue (CBB) staining as described below. The results showed that the purity of the influenza A and B viruses was over 99%.

### 2.5. Measurement of TCID_50_ (Tissue Culture Infectious Dose 50)

Viral titers were determined by performing 10-fold serial dilutions of samples in 96-well plates containing Madin-Darby Canine Kidney (MDCK) cells with 8 replicates of each sample. Before infection, cells were washed with phosphate-buffered saline (PBS). Infected cells were incubated at 37°C with 5% CO_2_, and fresh trypsin was added (final concentration, 0.03%). Viral titers were read as TCID_50_ calculated by the method of Reed and Muench [12].

### 2.6. Immunochromatography for Influenza A and B Viruses

The amount of influenza A and B viruses nucleoprotein (NP) in the collected solution was analyzed by immunochromatography (ESPLINE Influenza A & B-N, Fujirebio Inc., Tokyo, Japan) as follows. Briefly, the immunochromatography test assembly was developed using anti-influenza virus and anti-immunoglobulin (Ig) antibodies. The anti-influenza A and B NP antibodies were immobilized onto a nitrocellulose membrane for test lines (lines A and B, resp.) to capture influenza virus NP. To prepare the reference line, an anti-Ig antibody was immobilized onto a nitrocellulose membrane to capture Ig. A conjugated pad containing anti-influenza virus NPs used for the test line was labelled with colloidal gold, impregnated onto glass fibers, dried, and then placed between the test lines and the sample dropping region. The nitrocellulose membrane and glass fiber pad were assembled with a glass fiber pad on a plastic sheet within a plastic case. This assembled kit was stored in a bag with desiccant at 4°C until required. The kit was then used to quantify the amount of influenza virus NPs.

### 2.7. Polymerase Chain Reaction (PCR) for Influenza Virus

Viral genomic RNA was extracted from untreated and N_2_ gas plasma treated allantoic fluid infected with influenza A virus (A/PR/8/34) using a QIAamp Viral RNA mini kit (Qiagen, Hilden, Germany). The RNA was transcribed with PrimeScriptII 1st strand cDNA Synthesis kit (Takara Bio Inc., Otsu, Japan) and random primers to make cDNA by using the following temperature regime: 65°C for 5 min, 4°C for 5 min, and 42°C for 60 min. The resultant cDNAs were subjected to PCR for matrix protein (M1), hemagglutinin (HA), neuraminidase (NA), and nonstructural protein (NS) using Takara Ex Taq (Takara Bio Inc.). The temperature cycling conditions used for the PCR were 95°C for 5 min, 25 cycles of 95°C for 1 min, 55°C for 1 min, and 72°C for 1 min with one final cycle of 72°C for 10 min. PCR was carried out using the following primers modified from previous papers [13, 14]: MA1-F: 5′-CAG AGA CTT GAA GAT GTC TTT GCT G-3′; MA1-R: GCT CTG TCC ATG TTA TTT GGA TC-3′; HA-F: 5′-AGC AAA AGC AGG GGA AAA TAA-3′; HA-R: 5′-GCT ATT TCT GGG GTG AAT CT-3′; NA-F: 5′-TTG CTT GGT CGG CAA GTG C-3′; NA-R: 5′-CCA GTC CAC CCA TTT GGA TCC-3′; NS-F: 5′-AAG GGC TTT CAC CGA AGA GG-3′; NS-R: 5′-CCC ATT CTC ATT ACT GCT TC-3′. The intensity of the amplified bands from the PCR products was semiquantitatively analyzed by agarose gel electrophoresis. Bands in test samples were visually compared to those in untreated controls. The amplified PCR products generated from each pair of primers were verified by DNA sequencing.

### 2.8. Hemagglutination Assay

Samples were serially diluted two- or three-fold in 25 *μ*L of PBS in V-shaped well plates, and an equal volume of 1% chicken erythrocytes in suspension was added. The mixture was then incubated at room temperature for 1 h. The agglutination pattern was read, and the hemagglutination titer was defined as the reciprocal of the last dilution of sample that showed hemagglutination.

### 2.9. Enzyme Linked Immunosorbent Assay (ELISA) for Influenza Virus

A Serion ELISA antigen influenza B virus test (Institut Virion/Serion GmbH, Würzburg, Germany) was used for quantification of influenza B virus NP.

### 2.10. SDS-PAGE and CBB Staining

Samples were solubilized in an equal volume of 2× SDS gel-loading buffer [90 mM Tris-HCl (pH 6.8), 10% mercaptoethanol, 2% SDS, 0.02% bromophenol blue, and 20% glycerol], boiled for 5 min, and separated by SDS-PAGE (12% gel). The protein bands in the gel were visualized by CBB staining.

### 2.11. Scanning Electron Microscopy (SEM)

The influenza virus-infected allantoic fluids were air-dried on cover glass, treated with N_2_ gas plasma, and then fixed with 2% glutaraldehyde/0.1 M phosphate buffer (pH 7.4) overnight at 4°C. The cover glasses were subsequently treated with 2% osmium tetraoxide at 4°C for 3 h. Samples were dehydrated through a graded ethanol series (50–100% ethanol) at room temperature. Finally, the cover glass was subjected to critical point drying and evaporation coating by osmium plasma ion. SEM was then performed using a JSM-6320F (JEOL Ltd., Tokyo, Japan) instrument at a magnification of x50,000.

### 2.12. Influenza Virus Bioassay Using Embryonated Eggs

N_2_ gas plasma treated samples were injected into 11-day-old chicken embryonated eggs. The eggs were cultured at 37°C for 48 h before allantoic fluid was collected. The obtained samples were then subjected to immunochromatography for influenza virus and analyzed by hemagglutination assay.

### 2.13. Statistical Analysis

Results were compared by nonpaired Student's *t*-test. In cases where *P* < 0.05, the differences were considered significant.

## 3. Results

**Figure 2:**
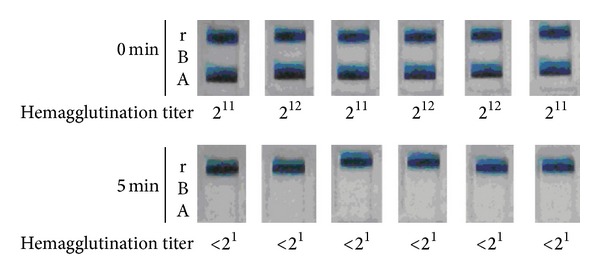
Inhibition of the proliferation of influenza virus in embryonated eggs by N_2_ gas plasma. Six independent spots of influenza A virus (A/PR/8/34) infected allantoic fluid (viral titer: 3.16 × 10^14^ TCID_50_/mL) on cover glass were air-dried, treated with N_2_ gas plasma (1.5 kpps, 5 min) using BLP-TES No.1, and injected into 6 embryonated chicken eggs. The eggs were incubated for 48 h before collecting allantoic fluid. The presence of influenza virus in the allantoic fluid was analyzed by immunochromatography for influenza virus NP (ESPRINE Influenza A & B-N, Fujirebio, Inc.). In addition, hemagglutination titers of each allantoic fluid were measured (see below). A and B indicate the lines for NP of influenza A virus and influenza B virus, respectively. The reference line (r) is also indicated. N_2_ gas plasma treatment (1.5 kpps) effectively inactivates influenza virus within 5 min.

**Figure 3:**
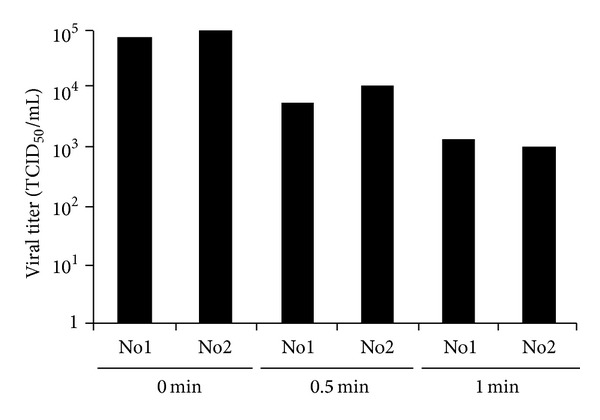
Decrease in viral titer of influenza virus by N_2_ gas plasma. Influenza A virus (A/PR/8/34)-infected allantoic fluid on a cover glass was air-dried, treated with N_2_ gas plasma (1.5 kpps; 0, 0.5, and 1 min) using BLP-TES No.1, and incubated with MDCK cells. After 72 h incubation, viral titers of TCID_50_/mL were calculated as described in Section 2. The assay was done in duplicate (No 1 and No 2), which was derived using different dried spots.

First, we investigated the N_2_ gas plasma treated influenza virus (A/PR/8/34) in infected allantoic fluid and determined whether influenza virus was inactivated by N_2_ gas plasma treatment (Figure 2). Samples including influenza virus (A/PR/8/34) at 3.16 × 10^14^ TCID_50_/mL were treated with N_2_ gas plasma for 5 min and injected into 11-day-old chicken embryonated eggs. After 48 h incubation at 37°C, the allantoic fluids were subjected to immunochromatography for influenza A virus to check whether the N_2_ gas plasma treated influenza virus had proliferated in the embryonated eggs. Influenza viruses derived from all six independent spots treated with N_2_ gas plasma for 5 min were unable to proliferate. By contrast, influenza viruses derived from six untreated spots did proliferate all. Morever, these results were consistent with those obtained from the hemagglutination assay. In addition, an infection assay using MDCK cells showed that viral titers of TCID_50_/mL changed from 7.5 × 10^4^ and 10 × 10^4^ at 0 min to 5.6 × 10^3^ and 10 × 10^3^ at 0.5 min and 1.3 × 10^3^ and 1.0 × 10^3^ at 1 min (Figure 3). These results also supported the inactivation of influenza virus by N_2_ gas plasma.

**Figure 4:**
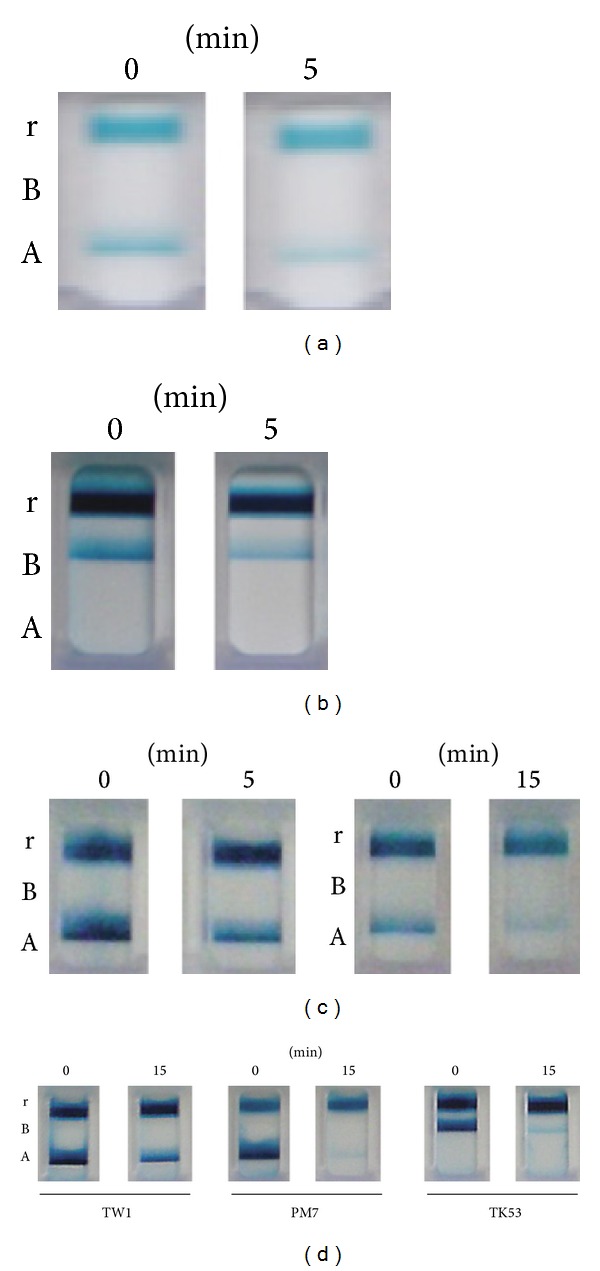
Effects of N_2_ gas plasma on nucleoprotein (NP) of influenza virus types A and B. Influenza A (a) and influenza B (b) viruses in nasal aspirate, influenza A virus (A/PR/8/34) in allantoic fluid (Viral titer: 3.16 × 10^14^ TCID_50_/mL) (c), and purified influenza viruses (d) were used. The samples were air-dried on cover glass and subjected to N_2_ gas plasma treatment (1.5 kpps) for the indicated time (min) using BLP-TES No.1. Samples were subsequently recovered in distilled water and then analyzed by immunochromatography (ESPRINE Influenza A & B-N, Fujirebio, Inc.). A and B indicate the lines for NP of influenza A virus and influenza B virus, respectively. The reference line (r) is also indicated. Influenza A virus H1N1 strain TW1: A/Taiwan/1/86 and H3N2 strain PM7: A/Panama/2007/99 were used. Influenza B virus TK53: B/Tokio/53/99 was also used.

Next, the effect of N_2_ gas plasma on viral proteins was investigated. The results from immunochromatography show that NP of influenza A and B viruses was decomposed by N_2_ gas plasma treatment of (i) nasal aspirates for 5 min (Figures 4(a) and 4(b)), (ii) allantoic fluid for 5 min or 15 min (Figure 4(c)), and (iii) purified virus for 15 min (Figure 4(d)). Specifically, a band corresponding to NP in the test line A and B was detected at 0 min, but it became less obvious after N_2_ gas plasma treatment for 5 or 15 min. A band in the reference line was detected at all time points indicating that the immunochromatography was working as anticipated. Regarding the nasal aspirates, all 3 influenza A and 3 influenza B samples showed a similar tendency. Based on this result we conclude that NP in the nasal aspirates and allantoic fluid was decomposed by N_2_ gas plasma treatment.

**Figure 5:**
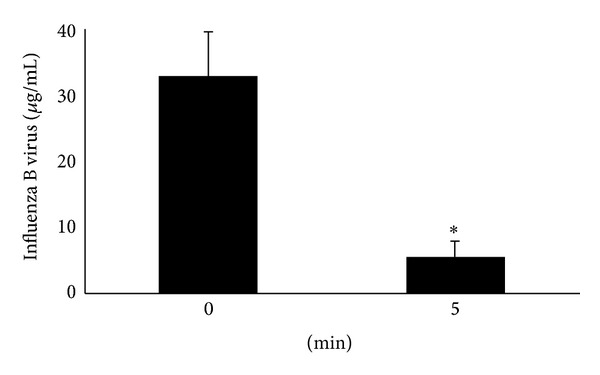
Degradation of viral proteins in influenza virus by N_2_ gas plasma. Quantity of influenza B virus (GF21: B/Gifu/2/73) derived from infected allantoic fluid before and after treatment with N_2_ gas plasma. Allantoic fluid (20 *μ*L) was dried on a cover glass and treated with N_2_ gas plasma at 1.5 kpps for 0 or 5 min. The quantity of NP of influenza B virus was measured by an enzyme-linked immunosorbent assay against NP. Asterisk indicates a significant difference (*P* < 0.05) when compared with 0 min.

Next, ELISA using anti-influenza B virus NP antibody was carried out to verify degradation of NP in influenza B virus derived from infected allantoic fluid that had been treated with N_2_ gas plasma (Figure 5). Within 5 min, the concentration of influenza B virus NP was decreased to less than 1/6 for B/Gifu/2/73. These results are consistent with previous and present immunochromatography findings regarding NP of influenza A and B viruses, which was shown to be degraded by the N_2_ gas plasma [15].

**Figure 6:**
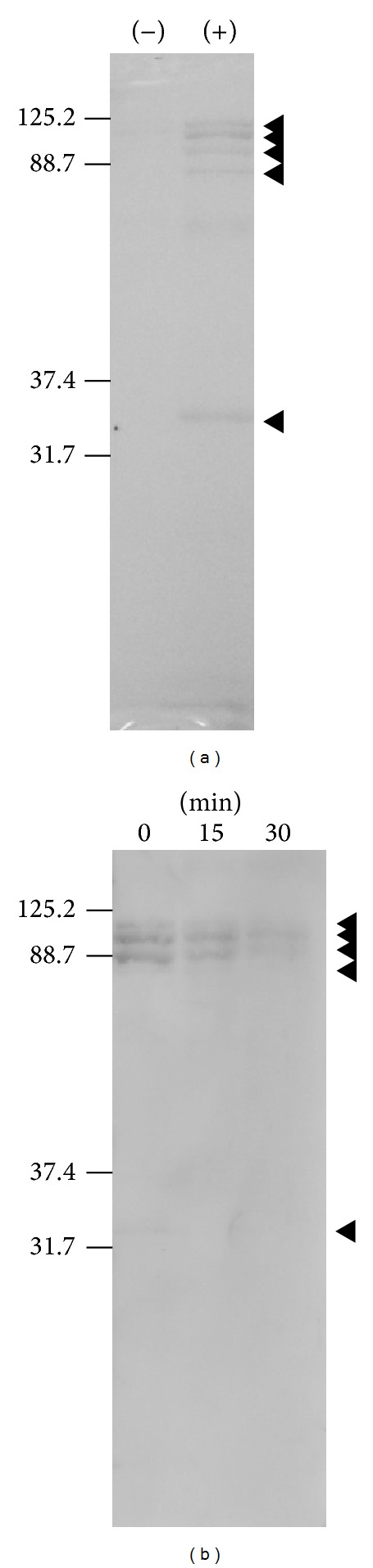
Degradation of influenza virus-infection induced proteins by N_2_ gas plasma treatment. (a) Proteins in influenza virus (A/PR/8/34) infected (+) and uninfected (−) allantoic fluid were subjected to sodium dodecyl sulfate (SDS) polyacrylamide gel electrophoresis (PAGE) and stained with Coomassie brilliant blue (CBB) solution. (b) The level of influenza virus-infection induced proteins in allantoic fluid, which include both viral proteins and the other proteins in allantoic fluid responding to the infection, was decreased after N_2_ gas plasma treatment for 15 and 30 min compared to the untreated control (0 min). The arrowheads highlight protein bands induced after influenza virus infection. The positions of the molecular weight markers are indicated on the left hand side.

SDS-PAGE analysis followed by CBB staining was also used to monitor viral proteins and/or induced proteins in the allantoic fluid after infection with influenza A virus (A/PR/8/34) (Figure 6(a)). Our results show that the proteins were degraded after N_2_ gas plasma treatment (1.5 kpps) for either 15 or 30 min (Figure 6(b)). A previous study reported that the major viral proteins in influenza virus derived from allantoic fluid are NA oligomer (over 70 kDa), HA1 (around 70 kDa), NA monomer and NP (around 70 kDa), and M1 and HA2 (around 30 kDa) [16]. Similarly sized bands appeared to be detected in the present SDS-PAGE and CBB stained gels of virus-infected allantoic fluid but faded after treatment of the fluid with N_2_ gas plasma. Therefore, the bands observed and degraded by N_2_ gas plasma may be mainly influenza viral proteins.

**Figure 7:**
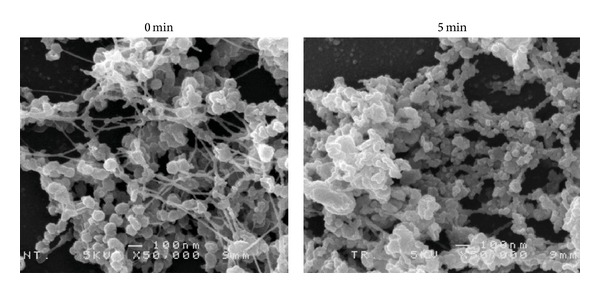
Change in morphology of influenza virus after treatment with N_2_ gas plasma. Influenza A virus (A/PR/8/34) in allantoic fluid was air-dried on cover glass and treated with N_2_ gas plasma (1.5 kpps for 5 min) using BLP-TES No.1. An identical sample was also prepared that was not treated with N_2_ gas plasma. The morphologies of influenza virus were observed at 5 kV with a magnification of x50,000 by SEM (JSM-6320F, JEOL Ltd.).

Next, morphologies of the influenza viruses were observed by SEM (Figure 7). Our SEM observations showed that the N_2_ gas plasma treatment (1.5 kpps, 5 min) disrupted fibers connecting influenza viruses in the allantoic fluid. Moreover, the N_2_ gas plasma treated influenza viruses displayed a shrunken appearance. In addition, fused viruses were also observed in the treated samples, suggesting that the N_2_ gas plasma may modify the viral envelope.

**Figure 8:**
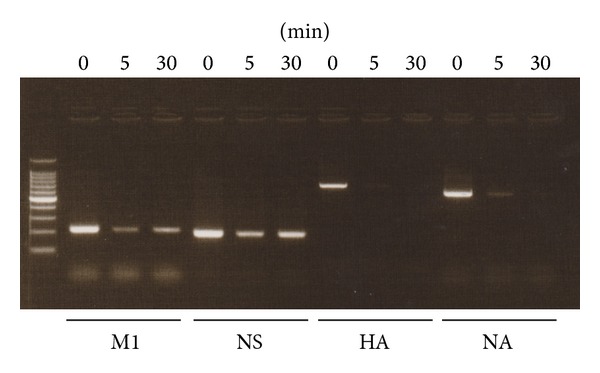
N_2_ gas plasma treatment causes damage to viral genomic RNA. An aliquot of influenza A virus (A/PR/8/34) infected allantoic fluid was dropped onto a cover glass and air-dried. The dried spot was then treated with N_2_ gas plasma (1.5 kpps) for the indicated times. The viral genomic RNA of the influenza virus was subsequently extracted and subjected to reverse transcription in order to generate complementary DNA (cDNA). Damage to the genomic RNA of influenza virus induced by the N_2_ gas plasma treatment was analyzed using the polymerase chain reaction (PCR). Namely, PCR was performed using cDNAs as template and specific sets of primers for matrix protein (M1), nonstructural protein (NS), hemagglutinin (HA), and neuraminidase (NA) of influenza virus. DNA size marker (100 bp DNA ladder Dye plus, Takara Bio Inc.) was run on the left hand lane of the gel.

Next, the N_2_ gas plasma treated influenza viruses were subjected to viral genomic RNA extraction. We then attempted to amplify various influenza virus genes, such as M1, NS, HA, and NA, by PCR (Figure 8). The results showed that amplification of each of the genes was greatly repressed by N_2_ gas plasma treatment (1.5 kpps; 5 and 30 min), suggesting that the viral genomic RNA was damaged following N_2_ gas plasma treatment. The magnitude of the observed decrease in the amplified product varied among the different viral genes. The inhibition efficiency of HA and NA was high compared to that of M1 and NS. This may be due to the structural differences and arrangements of influenza virus segments encoding each viral gene in the viral particle. The products amplified for M1 and NS appeared to be slightly more intense after 30 min of treatment than those observed after 5 min of treatment, whereas the amplified products for NA appeared to be slightly more intense after 5 min of treatment than those observed after 30 min of treatment. Repeated experiments showed that the band intensities for M1, NS, HA, and NA at 5 min and 30 min of treatment were similar.

## 4. Discussion

Influenza virus can be relatively easily disinfected by chemicals such as alcohol [17, 18]. The presence of organic matter interferes with the action of chemical sterilants because they can react, thereby decreasing the efficiency of disinfection. Indeed, disinfection efficiency of alcohol against influenza virus varies depending on the presence of coexisting organic material [19]. Most regulatory authorities require sterilant efficacy testing to be conducted in the presence of 5% soil [20]. Influenza virus is usually encountered in the nasal fluid of patients and allantoic fluid of eggs. In the case of medical devices, bronchoscopes in particular may be at risk of influenza virus contamination. In our study, we used 100% nasal aspirates and 100% allantoic fluid as a source of organic material. The obtained results showed that N_2_ gas plasma can inactivate influenza virus in these environments. Currently, the items/areas that can be treated by the method are restricted to the size of the chamber box of the N_2_ gas plasma instrument. To enable the sterilization of medical devices and larger surfaces, it would be necessary to expand the size of the discharge area.

NP and genomic RNA, which are both localized to the central region of the influenza virus particle, were subject to decomposition and/or modification by treatment with N_2_ gas plasma. Likewise, lipids localized in the outer envelope of the virus were also modified and/or degraded after this treatment. Microscopic investigations showed that treatment with N_2_ gas plasma disrupt the fibers between virus particles in the allantoic fluid, although the significance of the fibers connecting the viruses in the untreated samples in relation to the infectivity of the virus is unclear. Previous studies have shown that oxidative stress contributes to the mechanism of action of a gas plasma. For example, the addition of oxygen to helium has been found to enhance the efficiency of inactivation in the case of bacteria [21]. In addition, oxidation and peroxidation processes on the surface of cells and within cells result in inactivation [22, 23]. Furthermore, destruction of the surface structure by gas plasma may be the main mechanism underlying the inactivation of bacteria [24], which may also be the case for viruses. Although these oxidative factors contribute to the mechanism of action in N_2_ gas plasma, further studies are required to identify the most critical factor(s) for inactivation.

In this study, we analyzed the effect of N_2_ gas plasma treatment on influenza virus, which is enveloped. However, the effectiveness of this treatment against various other pathogens, which may differ in resistance to disinfection, is unknown. For example, it would be interesting to investigate the effect of this treatment on nonenveloped viruses, such as norovirus or adenovirus as well as the highly resistant prion agents.

## 5. Conclusion

In conclusion, the present results suggest that N_2_ gas plasma treatment modifies viral genomic RNA and degrades viral proteins, including NPs, as well as the viral envelope and fibers related to allantoic fluid. Taken together, the results indicate that N_2_ gas plasma treatment may be an effective means of disinfection for influenza virus.
